# Prevalence, Prevention, and Lifestyle Intervention of Gestational Diabetes Mellitus in China

**DOI:** 10.3390/ijerph17249517

**Published:** 2020-12-18

**Authors:** Juan Juan, Huixia Yang

**Affiliations:** Department of Obstetrics and Gynecology, Peking University First Hospital, Beijing 100034, China; juanjuan@bjmu.edu.cn

**Keywords:** gestational diabetes mellitus, prevalence, risk factor, prevention and lifestyle intervention, China

## Abstract

Gestational diabetes mellitus (GDM) has become an epidemic and has caused a tremendous healthy and economic burden in China, especially after the “two-child policy” put into effect on October 2015. The prevalence of GDM has continued to increase during the past few decades and is likely to see a further rise in the future. The public health impact of GDM is becoming more apparent in China and it might lead to the development of chronic non-communicable diseases in the long-term for both mothers and their children. Early identification of high-risk individuals could help to take preventive and intervention measures to reduce the risk of GDM and adverse perinatal outcomes. Therefore, a focus on prevention and intervention of GDM in China is of great importance. Lifestyle interventions, including dietary and physical exercise intervention, are effective and first-line preventive strategies for GDM prevention and intervention. The GDM One-day Care Clinic established in 2011, which educates GDM patients on the basic knowledge of GDM, dietary intervention, physical exercise, weight management, and blood glucose self-monitoring methods, sets a good model for group management of GDM and has been implemented throughout the hospitals as well as maternal and child health centers in China. The current review focus on the prevalence, risk factors, as well as prevention and lifestyle intervention of GDM in China for better understanding of the latest epidemiology of GDM in China and help to improve maternal and neonatal pregnancy outcomes and promote long-term health for women with GDM.

## 1. Background

Gestational diabetes mellitus (GDM) is one of the common metabolic complication during pregnancy and is associated with an increased risk of adverse pregnancy outcomes for both mothers and their offspring not only in the short-term but also in the long-term [1,2,3,4]. Women with GDM are at an increased risk of perinatal morbidity. These women are also at particular high risk of diabetes and cardiovascular disease in their later life. According to a meta-analysis of 20 studies and 1,332,373 individuals (67,956 women with GDM and 1,264,417 controls), women with a history of GDM have a nearly 10-fold higher risk of developing type 2 diabetes mellitus (T2DM) than those with normoglycemia during pregnancy [3]. Offspring born to women with GDM are at an increased risk of macrosomia, neonatal hypoglycemia, hyperbilirubinemia, and neonatal respiratory distress syndrome, as well as obesity in childhood and cardiovascular disease traits in adulthood [1]. There has been a marked increase in the prevalence of GDM in China over the last few decades [5]. The increased prevalence of GDM has caused a huge societal economic and healthy burden at both the population and individual levels for China. A modelling study showed that on average, the cost of a pregnancy with GDM was ¥6677.37 more than a pregnancy without GDM, due to additional expenses during both the pregnancy and delivery in 2015 [6]. After the “two-child policy” fully implemented in China on October 2015, the prevalence of high-risk pregnant women, including elder age, pre-pregnancy overweight or obesity, has been risen dramatically and it is likely to see further increase in the GDM prevalence, giving rise to a tremendous burden on the healthcare system in China. The public health impact of GDM is becoming more apparent, as it is a strong predictor of the development of chronic non-communicable diseases in the future [7]. The current review focuses on the prevalence, risk factors, prevention, and lifestyle intervention of GDM in China to better understand the latest epidemiology of GDM in China, to help improve maternal and neonatal pregnancy outcomes, and promote long-term health for women with GDM.

## 2. Prevalence of GDM in China

Globally, the prevalence of GDM has continued to increase during the past few decades [8]. According to the International Diabetes Federation (IDF), GDM occurs in approximately 14% globally, ranging from 9% in Africa, 12.6% in North America and 21% in Asia in 2017 [9]. A meta-analysis investigating the GDM prevalence in Eastern and Southeast Asia showed that the incidence of GDM in China was reported to be 11.91%, which was much higher than Japan, Korea, and Thailand, with a GDM prevalence of less than 8.0% [10]. As a result of economic development, and improvements in living standards, together with lifestyle changes with increasing westernization, characterized by changes in dietary patterns and physical inactivity, together with increased attention to GDM screening, China has witnessed a dramatic rise in the prevalence of GDM. The prevalence of GDM increased almost 3.5-fold from 1999 to 2012 according to the data of universal screening for GDM in Tianjin, China (Table 1) [11,12,13]. In 2011, after the launch of the landmark International Association of Diabetes and Pregnancy Study Group (IADPSG) diagnostic criteria based on the dose-response association between increasing maternal glucose levels and adverse maternal, fetal, and neonatal outcomes reported by the Hyperglycemia and Adverse Pregnancy Outcome (HAPO) study [14], many international organizations, including the World Health Organization (WHO) [15], American Diabetes Association (ADA) [16], and Federation International of Gynecology and Obstetrics (FIGO) [17], have adopted this as the new diagnostic criteria for GDM. China also revised the GDM diagnostic criteria according to IADPSG criteria to be in line with international standards. After the new diagnostic criteria was carried out in China, the prevalence of GDM was largely increased as more pregnant women with mild hyperglycemia are diagnosed as GDM by the new criteria. By 2013, the prevalence of GDM was up to 19.7% among 15,194 pregnant women in 15 hospitals in Beijing, China according to the new criteria of IADPSG [18]. The latest systematic review and meta-analysis including 25 cross-sectional studies or retrospective studies and 79,064 Chinese participants showed that the pooled prevalence of GDM in mainland China according to IADPSG criteria was 14.8% (95% confidence interval (CI): 12.8–16.7%) [19]. Although the prevalence of GDM was different depending on different diagnostic criteria, it showed an upward trend during the past few years.

The prevalence of GDM varies across different cities and regions of China, as China encompasses a vast territory, and has a large population with differences in regions, ethnicities, diets, and living habits (Table 1). The prevalence of GDM varied from high to low in the east, south, west, northwest portions in China, respectively. The eastern and southern parts of China, characterized by relatively better economic development, have a much higher prevalence of GDM. A study according to 27,119 pregnant women in Tongzhou district of Beijing during 2013–2018 showed that the overall prevalence of GDM was 24.24% according to the IADPSG criteria [21]. The higher prevalence in Tongzhou district might due to the traditional dietary and lifestyle of individuals in this suburb area of Beijing, as they still lack of knowledge on how to keep a balanced diet and healthy lifestyle during pregnancy, which raised an urgent need for education on healthy diet and lifestyle around China. A recent study in Eastern China, Qingdao also showed an exceptionally high estimated prevalence of GDM of 21.8% according to IADPSG criteria in 2016 among three hospitals through randomly stratified and cluster sampling selection [22]. Another study conducted in Linyi maternity hospital in Shandong province reported a similar prevalence of 21.82% based on IADPSG criteria. In Southern China, a retrospective study conducted in the First Affiliated Hospital of Jinan University in Guangdong China from 2011–2017 demonstrated that the prevalence of GDM was 22.94% [23]. A linked-database cohort study used the Medical Birth Registry of Xiamen among 78,572 women between 2011 and 2018 showed that GDM affects 17.6% of all pregnant women in Xiamen [24]. In Western China, the age-standardized prevalence of GDM was 18.3% (95% CI: 15.6–21.1%) in Chengdu, Sichuan province [25]. However, in some parts of China, which might due to the limitations of adequate resources and education, the prevalence of GDM is relatively low, such as in Northwestern China, the prevalence of GDM was only 5.12% in Xinjiang according to IADPSG criteria in 2013 [26]. The reasons for the wide variation are unclear, but may be because of the differences in the characteristics of participants in different studies (such as, age, family history of diabetes, physical activity, and dietary habits) as well as differences in study settings and economic situation [19].

Though the diagnostic criteria of GDM continues to be debated and has been changed continuously since 1990s in China, nowadays, the most acceptable diagnosis criteria among medical institutes across China is based on the landmark IADPSG diagnostic criteria launched in 2011. After the launch of the new IADPSG diagnostic criteria according to the HAPO study [14], several studies have conducted to evaluate the suitability of the new IADPSG criteria of GDM in China. A retrospective study of 14,593 pregnant women was conducted and the results showed that the incidence of perinatal complications increased in pregnant women who met the IADPSG criteria without interventions, and indicated that the IADPSG diagnostic criteria were suitable in China [27]. In 2014, another prospective study conducted among 25,674 pregnant women in Peking University First Hospital reported that treatment or intervention of women with GDM identified by IADPSG criteria were significantly associated with lower risk of multiple adverse pregnancy outcomes. These findings provided more support for applying the IADPSG criteria in China [28]. Therefore, China also revised the GDM diagnostic criteria according to IADPSG criteria to be in line with international standards. The Ministry of Health in China formulated the “Diagnostic Criteria for Gestational Diabetes Mellitus (WS 331-2011)” [29,30] and recommended performing a diagnostic 75 g OGTT during 24–28 weeks of gestation for all pregnant women who were not previously diagnosed with overt diabetes. This one-step approach could be applied to well-resourced medical institutions. On the other hand, a two-step approach was recommended if it is difficult to implement a formal 75 g OGTT in low-resourced rural areas of China. To further standardize the diagnosis of GDM and promote a universal diagnostic guideline in different regions of China, the Obstetrics and Gynecology Branch of Chinese Medical Association and the Perinatal Medicine Branch of Chinese Medical Association jointly developed the “Guideline for Diagnosis and Treatment of Gestational Diabetes Mellitus (2014)” [31]. This updated guideline referred to the IADPSG diagnostic criteria of GDM [14], the GDM health industry standards issued by the Ministry of Health in China in 2011 [29,30], and guidelines published by international institutes and countries. Nowadays the guideline has gained acceptance among medical institutes across China. The current diagnostic criteria of GDM used in China are a 2-h 75 g OGTT performed during 24–28 gestational week in all pregnant women without overt diabetes. The GDM could be diagnosed if any of the OGTT plasma glucose values meets or exceeds the following cutoff values: 5.1 mmol/L at fasting; 10.0 mmol/L at 1 h; and 8.5 mmol/L at 2 h. Although a 75 g OGTT is required to detect all women with GDM, due to the complexities of Chinese vast geography and population, this procedure is not feasible in many low-resourced medical clinics in China. In this circumstance, Fasting Plasma Glucose (FPG) during 24–28 weeks of gestation could be done as an acceptable alternative approach to 75 g OGTT and only for those with 4.4 mmol/L ≤ FPG < 5.1 mmol/L, a 75 g OGTT is needed for the diagnosis of GDM. The guidelines for diagnosis of GDM in China are shown in Table 2.

## 3. Risk Factors for GDM in China

The rising prevalence of GDM in China is partly due to the concurrent increases in well-established risk factors, such as advanced maternal age, pre-pregnancy overweight or obesity, excessive gestational weight gain [13]. After the “two-child policy” put into practice in China in October 2015, the incidence of high-risk pregnant women of GDM, including women who had a history of GDM, has been largely increased. Early identification of risk factors of GDM could help to take preventive measures to avoid developing GDM and adverse perinatal outcomes. Well-known risk factors for GDM are listed in Table 3.

### 3.1. Advanced Maternal Age

Advanced maternal age is a strong risk factor for GDM, especially for women with maternal age of 35 years or older. The incidence of GDM in women with advanced age was 26.7% (95% CI: 23.2–30.2%), whereas in younger pregnant women, the incidence was only 13.4% (95% CI: 11.0–15.7%), with a significant difference between the two subgroups (*p* < 0.01) [19]. Insulin sensitivity and pancreatic β-cell function reduced in pregnant women of elder age, which increased the risk of glucose and lipid metabolism abnormal during pregnancy. A systematic review and meta-analysis of cohort studies with over 120 million participants demonstrated that for women aged ≥ 40 years, 35–39 years, 30–34 years, 25–29 years and < 20 years, the ORs and 95% CIs were 4.86 (95% CI: 3.78–6.24), 3.54 (95% CI: 2.88–4.34), 2.73 (95% CI: 2.28–3.27), 1.69 (95% CI: 1.49–1.93), and 0.60 (95% CI: 0.50–0.72), respectively. The risk of GDM exhibited a linear relationship with maternal age according to dose-response analysis (*P*_trend_ < 0.001). For each one-year increase in maternal age from 18 years, the risk of GDM increased by 12.74% for Asian women [34]. In Western China, the incidence of GDM also increased with advanced maternal age, the incidence was 23.7%, 15.7%, and 12.6% among women who were aged ≥30 years, 25–29 years, and <25 years, respectively [25]. In Eastern China, a similar trend was also witnessed, the incidence of GDM was 32.1%, 14.8%, and 9.7% among those aged ≥30 years, 25–29 years, and <25 years, respectively [22]. According to a cross-sectional study of 16 hospitals from five selected provinces in mainland China, women aged 36–45 years had a nearly 4-fold risk of having GDM compared to those with 18–25 years old (OR = 3.98, 95% CI: 1.41–11.28) [35]. Another prospective cohort study of the Born in Guangzhou Cohort Study in China showed similar results that women older than 35 years had a 3.95-fold increased risk of GDM (95% CI: 2.80–5.58) compared to women aged 16–25 years [49].

### 3.2. Pre-Pregnancy Overweight or Obesity

Pre-pregnancy body mass index (BMI) is commonly used in risk factor screening for GDM and pre-pregnancy overweight or obesity is an established risk factor for GDM, which can significantly increase the incidence of GDM [22,50]. The risk of GDM increases with increasing pre-pregnancy BMI. Among Asian women, the prevalence was highest among women with BMI ≥ 30 kg/m^2^ (13.78%), followed by BMI ≥ 25 kg/m^2^ (10.22%) and then BMI ≥ 20 kg/m^2^ (6.09%) [36]. A similar upward trend was also evident for the prevalence of GDM in Western and Eastern China in relation to pre-pregnancy BMI, in which the incidence was 27.9%, 18.4%, and 10.8% among overweight, normal weight, and underweight individuals, respectively in Western China [25] and 45.7%, 17.8%, and 6.0% among those with pre-pregnancy BMI ≥ 24 kg/m^2^, 18.5–23.9 kg/m^2^, and < 18.5 kg/m^2^, respectively, in Eastern China [22]. A meta-analysis of 84 studies in Asia found that pre-pregnancy BMI ≥ 25 kg/m^2^ could increase the risk of GDM by more than three-fold (OR = 3.27, 95% CI 2.81–3.80) [37].

### 3.3. Excessive Gestational Weight Gain

Gestational weight gain is one of the major and modifiable risk factors for GDM. High rates of gestational weight gain, particular during early pregnancy, may increase the risk of GDM. Compared with lower rate of gestational weight gain (less than 0.28 kg per week), a rate of weight gain of 0.28 kg per week or more was associated with an increased risk of GDM (OR = 2.03, 95% CI: 1.15–3.59) according to a study conducted in Peking University First Hospital in China [38]. Another prospective study from Southwest China showed that gestational weight gain in the first trimester above IOM recommendations increased the risk of GDM among underweight (OR = 2.50, 95% CI: 1.11–5.66), normal-weight (OR = 1.40, 95% CI: 1.02–1.91), and overweight/obese women (OR = 3.02, 95% CI: 1.12–8.14) compared with those within IOM recommendations, which indicated that excessive gestational weight gain in the first trimester is associated with increased risk of GDM regardless of pre-pregnancy BMI [51].

### 3.4. History of GDM

Having a previous pregnancy complicated by GDM is one of the major contributing risk factors for the development of GDM in the subsequent pregnancy. It has been estimated that GDM occurs in about 30–69% of subsequent pregnancies after an initial pregnancy affected with GDM [43,44,52]. A meta-analysis of 84 studies in Asia showed that pregnant women with history of GDM were more likely to develop GDM compared to those without history of GDM.

### 3.5. Changes in Dietary Pattern and Lifestyle

China has undergone marked economic improvement in recent decades. Increasing globalization and East-West exchanges has led to changes in dietary patterns and lifestyles as well as promoted overnutrition and sedentary lifestyles. A systematic review and meta-analysis of longitudinal or cohort studies with 30,871 pregnant women demonstrated that frequent consumption of potato, meat/processed meat, and protein (% energy) derived from animal sources were associated with an increased risk of GDM [39]. Evidence from the Chinese Prospective Birth Cohort Study showed that dietary pattern with high protein intake, characterized by fish, meat, and eggs, was associated with a higher risk of GDM [40]. Another prospective cohort study in Northern China showed that compared with prudent pattern (dark-colored vegetables and deep-sea fish), both the Western pattern (dairy, baked/fried food and white meat) (OR = 4.40, 95% CI: 1.58–12.22) and the traditional pattern (light-colored vegetables, fine grain, red meat and tubers) (OR = 4.88, 95% CI: 1.79–13.32) during pregnancy were associated with an increased risk of GDM [41]. Additionally, prospective cohort study in China demonstrated that sweets and seafood pattern was independently associated with an increased risk of GDM (RR = 1.23, 95% CI: 1.02–1.49) [53]. Sedentary lifestyle has also been reported to be a risk factor of GDM among Chinese pregnant women. A population-based cross-sectional study in Tianjin, China reported that sitting at home for 2–4 h per day (OR = 1.59, 95% CI: 1.18–2.15) and >4 h per day (OR = 1.73, 95% CI: 1.22–2.43) were associated with significantly increased risk of GDM [50].

### 3.6. Family History of Diabetes in First-Degree Relatives

Family history of diabetes in first-degree relatives is independently associated with an increased risk of developing GDM. Several studies conducted in China have shown that pregnant women with a family history of diabetes in first-degree relatives were at 1.48–3.60 times higher risk of developing GDM than those without the history [18,22,42,54]. A study of 15,194 pregnant women conducted in 15 hospitals in Beijing showed that family history of diabetes is related to the risk of GDM (OR = 1.48, 95% CI: 1.254–1.748, *p* < 0.01) [18]. A cross-sectional study conducted in Qingdao, China including 4959 women showed that GDM was positively associated with both paternal history of diabetes (OR = 2.54, 95% CI = 1.38–4.67, *p* = 0.003;) and maternal history of diabetes (OR = 3.60, 95% CI = 2.16–5.98, *p* < 0.001) [22]. A retrospective case-control study of 3608 pregnant women reported that compared with those without a family history of diabetes, those with a family history of diabetes were 2.5 times more likely to develop GDM (*p* < 0.001) [50]. A matched pair case-control study with 276 GDM women and 276 non-GDM women conducted in two hospitals in Beijing, China demonstrated that family history of diabetes in first-degree relatives was significant risk factors for GDM in Chinese women (OR = 3.07, *p* = 0.004) [54].

### 3.7. Polycystic Ovarian Syndrome

Polycystic ovarian syndrome (PCOS) is a strong risk factor for GDM. In pregnant women with PCOS, both the physiologic state of hyperinsulinemic insulin resistance and early abnormal insulin action could worsen the insulin resistance, which significantly increase the risk of GDM [45]. A 1.5-fold increased risk for GDM in women with PCOS in early pregnancy was reported by a retrospective cohort study of 2389 Chinese pregnant women (OR = 1.55, 95% CI: 1.14–2.09) [46]. A Taiwan nationwide population-based study of 7629 women also showed that a history of PCOS is a significant and independent risk factor for development of GDM (OR = 2.15, 95% CI: 1.96–2.37) [47]. A cohort of Chinese women followed from their first diagnosis of PCOS to the birth of their neonate indicated that PCOS itself is a risk factor for developing GDM, independent of weight and age [55].

## 4. Prevention and Lifestyle Intervention of GDM in China

The concept of Developmental Origins of Health and Disease (DOHaD) proposed by David Barker has been widely accepted and according to this theory, the intrauterine environmental conditions in early life had profound implications for long-term health and the development of chronic non-communicable diseases in adulthood [56]. GDM provides unique opportunities for improving maternal and child health [57]. Numerous studies have shown that prevention and intervention of GDM could reduce the risk of perinatal and long-term diseases. Therefore, a focus on prevention and intervention of GDM in China is of great importance.

To face the increased prevalence and its very large economic and health burden in China after the adoption of the IADPSG diagnosis criteria in 2011, a comprehensive management model of GDM patients, the One-day Care Clinic [58,59], which including educate GDM patients on the basic knowledge of GDM, dietary intervention, physical exercise, weight management, and blood glucose self-monitoring methods by professional physicians and nurses, was established in May 2011 at Peking University First Hospital.

Lifestyle interventions, including dietary intervention and physical exercise, are effective and first-line preventive strategies for GDM prevention and intervention. It can also reduce the progression of high-risk individuals to GDM. All women are encouraged to keep good dietary and lifestyle habits during pregnancy. Though a European multi-centered randomized controlled trial (RCT), which enrolling consecutive pregnant women with a BMI ≥ 29 kg/m^2^, demonstrated that a healthy eating intervention combined with physical exercise resulted in less gestational weight gain, however with no impact on reducing fasting plasma glucose [60]. In contrast, the effectiveness of lifestyle intervention in early pregnancy for prevention of GDM has been supported by RCTs in Finnish and Chinese population [61,62,63]. The Finnish Gestational Diabetes Prevention Study (RADIEL) reported that a moderate individualized lifestyle intervention reduced the incidence of GDM by 39% in high-risk pregnant women [61]. The prospective randomized clinical trial conducted in China showed that physical exercise three times per week for at least 30 min per session started early in pregnancy could decreased the risk of GDM in overweight and obesity pregnant women by 45.8% [62,63]. A meta-analysis and meta-regression involving 47 RCTs and 15,745 participants showed that diet and exercise during pregnancy were preventive of GDM (RR = 0.77, 95% CI: 0.69–0.87) [64]. Another meta-analysis of 29 RCTs with 11,487 pregnant women reported that either diet or physical activity resulted in an 18% reduction in the risk of GDM (*p* = 0.009) [65]. Zhang et al. found that adherence to a low risk lifestyle (healthy body weight, healthy diet, regular exercise) was associated with a lower risk of GDM and could be an effective strategy for the prevention of GDM [66]. A systematic review and meta-analysis of RCTs about interventions to reduce the incidence of GDM reported that diet and lifestyle interventions reduced GDM risk by 62% (RR = 0.38, 95% CI: 0.24–0.59) and 32% (RR = 0.68, 95% CI: 0.54–0.86), respectively in Asia [67]. A cluster-randomized controlled study conducted in Peking University First Hospital reported that the incidence of GDM could be reduced by a certain amount lifestyle intervention, including education on “appropriated dietary, physical activity, and weight gain during pregnancy”, initiated from early in the first trimester [68].

Dietary intervention started during early pregnancy could significantly reduce the incidence of GDM [69]. According to a systematic review and meta-analysis of longitudinal or cohort studies with 30,871 pregnant women, diets such as Mediterranean Diet (MedDiet), Dietary Approaches to Stop Hypertension (DASH) diet and Alternate Healthy Eating Index diet (AHEI) were associated with 15–38% reduced relative risk of GDM [44]. A case-control study conducted in Taiyuan, China reported that a vitamin pattern diet (characterized as consumption of diet rich in vitamin A, carotene, vitamin B2, vitamin B6, vitamin C, dietary fiber, folate, calcium, and potassium) is associated with decreased risk of GDM in Chinese women [70]. Evidence from the Chinese Prospective Birth Cohort Study showed that dietary pattern with high vegetable, fruit, and rice intake was associated with a lower GDM risk among Chinese pregnant women [45]. Another study based on Tongji Maternal and Child Health Cohort demonstrated similar results that high carbohydrate intake (high rice-wheat-fruits scores) were associated with lower risk of GDM [71]. In addition, studies showed that plant-based dietary pattern may also lower the risk of GDM. Vegetable pattern was associated with a decreased risk of GDM (RR = 0.79, 95% CI: 0.64–0.97) [47]. It is indicated that a diverse diet based on plant and cereal food should be promoted for health and prevention of GDM during pregnancy. These findings might provide scientific basis for making dietary guidelines and recommendations among pregnant women in China to prevent GDM.

Physical exercise is a non-invasive therapeutic option for preventing and managing GDM. A prospective randomized clinical trial conducted in Peking University First Hospital, China showed that supervised stationary cycling three times per week for at least 30 min per session started early in pregnancy could decreased the risk of GDM in overweight and obesity pregnant women by 45.8% [61,62]. According to a population-based cross-sectional study in Tianjin, China, increased physical activity during pregnancy was associated with decreased risk of GDM (OR = 0.81, 95% CI: 0.67–0.97) among Chinese pregnant women [53]. A systematic review and meta-analysis of longitudinal or cohort studies with 30,871 pregnant women reported that any pre-pregnancy or early pregnancy physical activity compared to no physical activity was associated with 30% and 21% reduced odds of GDM, respectively. Engaging in >90 min/week of leisure time physical activity before pregnancy was associated with 46% decreased risk of GDM [44]. Another meta-analysis about practice intervention pointed out that physical activity during early pregnancy could help to reduce the incidence of GDM by about 24% (OR = 0.76, 95% CI: 0.70–0.83) [72]. Physical exercise during pregnancy is beneficial to both mothers’ and their fetuses’ health, including avoiding maternal excessive weight gain, maintaining fetal weight within the normal range, preventing pregnancy complications, and reducing the risk of macrosomia [73,74].

Therefore, systematic implementation of lifestyle intervention to increase physical activity and improve diet is key to GDM prevention efforts. However, despite an increase in leisure-time physical activity and the improvement of dietary behavior, the proportion of pregnant women affected by GDM in China continues to increase, suggesting more targeted interventions are needed for these high-risk individuals. Therefore, the GDM One-day Care Clinic has been implemented throughout the hospitals as well as maternal and child health care centers as part of the World Diabetes Foundation projects and set a good model for group management of GDM in China. However, more efforts are needed to promote its use on a larger scale.

In addition, women of reproductive age usually do not receive regular physical examination or know their blood glucose levels in China. Sometimes they do not notice that hyperglycemia is already present at conception [75]. Therefore, all women of reproductive age, especially for those in high-risk of diabetes and with a history of GDM, should seek preconception counseling before pregnancy, as well as evaluate their blood glucose levels and maintain glucose levels as close to normal as possible prior to conception. In addition, counseling on the specific risk factors for GDM is important and they should be educated on the lifestyle interventions to prevent GDM and other adverse perinatal outcomes [1].

Furthermore, keeping a healthy lifestyle and balanced diet is beneficial not only in the short-term, but also to the long-term health of the mother and their offspring [76]. Thus, more attention should be paid to postpartum follow-up of GDM patients and high-risk individuals to promote long-term health for both mothers and their children. However, the postpartum follow-up rate is relatively low in China currently, which might owe to the lack of self-efficacy and social support, as well as professional knowledge among health providers. Therefore, obstetricians and other healthcare providers should cooperate to provide more support for postpartum follow-up.

## 5. Conclusions

GDM has become an epidemic and caused a tremendous healthy and economic burden in China, especially after the “two-child policy” was put into effect in October 2015. The prevalence of GDM has continued to increase during the past few decades and is likely to see a further rise in the future. The rising prevalence of GDM in China is partly due to the concurrent increase in well-established risk factors, including advanced maternal age, pre-pregnancy overweight or obesity, excessive gestational weight gain, history of GDM, changes in dietary patterns and lifestyles, etc. The public health impact of GDM is becoming more apparent in China and it might lead to the development of chronic non-communicable diseases in the long-term for both mothers and their children. GDM provides unique opportunities for improving maternal and child health. Early identification of individuals of high risk could help to take preventive and interventive measures to reduce the risk of GDM and adverse perinatal outcomes. Therefore, a focus on prevention and intervention of GDM in China is of great importance. The diagnostic criteria of GDM in China has been developed since 1990s and now the most acceptable diagnostic criteria of GDM used in China are a 2-h 75 g OGTT performed during 24–28 gestational week according to the “Guideline for Diagnosis and Treatment of Gestational Diabetes Mellitus (2014)” based on the IADPSG diagnostic criteria. To face the increased prevalence and its large economic and health burden in China after the adoption of the IADPSG diagnosis criteria in 2011, a comprehensive management model of GDM patients, the One-day Care Clinic, which including educate GDM patients on the basic knowledge of GDM, dietary intervention, physical exercise, weight management, and blood glucose self-monitoring methods by professional physicians and nurses, was established in May 2011 at Peking University First Hospital and set a good model for group management of GDM as well as provide more targeted interventions in China. Lifestyle interventions, including dietary and physical exercise, are effective and first-line preventive strategies for GDM prevention and intervention. In addition, all women of reproductive age, especially for those in high-risk of diabetes or with a history of GDM, should seek preconception counseling before pregnancy, as well as evaluate blood glucose levels and maintain glucose levels as close to normal as possible prior to conception. They should also be educated on keeping healthy lifestyle and balanced diet for both pregnancy and postpartum. The identification of women at high risk for impaired postpartum glucose metabolism plays a crucial role in early initiation of effective interventional strategies to delay or prevent the progression from GDM to type 2 diabetes mellitus or other non-communicable diseases in both postpartum period and long term [77]. The guidelines of ADA recommended that women with GDM should be screened with 75 g OGTT at 4–12 weeks postpartum [1]. However, the postpartum follow-up rate is relatively low in China currently. According to a study conducted at Tianjin Obstetrics and Gynecology Hospital, only 13.1% women screened for blood glucose levels after delivery [78]. The lower rate might be attributed to a lack of social support and professional knowledge among health providers as well as some physicians and pregnant women not taking postpartum follow-up into account as part of their routine practice. Therefore, it is important to cooperate obstetricians, internists, pediatricians, and other healthcare providers to provide support as well as carry out postpartum follow-up and screening to identify women with high risk of type 2 diabetes and metabolic disorders later in life, and more pregnant women should be engaged in keeping healthy lifestyle postpartum to improve maternal and offspring health in the long term. Additionally, an integrated strategy combining population-wide preventive management, intensive health education, early detection, and multidisciplinary care programs should be strengthened for the prevention and control of GDM, especially in high-risk women, which could help to reduce the risk of GDM and associated complications in the general population and high-risk individuals and to improve maternal and neonatal pregnancy outcomes as well as promote long-term health for both mothers and their children.

## Figures and Tables

**Table 1:** The prevalence of GDM in different cities and regions of China.

City	Prevalence	Year	Diagnosis Criteria
Tianjin	2.3%	1999	WHO 1999 ^1^
Tianjin	6.8%	2008	WHO 1999
Tianjin	8.1%	2012	WHO 1999
Xinjiang	5.12%	2013	IADPSG 2011 ^2^
Beijing	19.7%	2013	IADPSG 2011
Qingdao, Shandong	21.8%	2016	IADPSG 2011
Linyi, Shandong	21.82%	2016	IADPSG 2011
Guangdong	22.94%	2017	IADPSG 2011
Xiamen, Fujian	17.6%	2018	IADPSG 2011
Tongzhou, Beijing	24.24%	2018	IADPSG 2011
Chengdu, Sichuan	18.3%	2019	IADPSG 2011

^1^ WHO 1999: GDM is diagnosed if a fasting glucose level of 75 g OGTT ≥ 7.0 mmol/L or a 2 h plasma glucose level of ≥11.1 mmol/L [20]. ^2^ IADPSG 2011: GDM is diagnosed if any of the 75 g OGTT plasma glucose values during 24–28 gestational weeks meets or exceeds the following cutoff values: 5.1 mmol/L at fasting; 10.0 mmol/L at 1 h; and 8.5 mmol/L at 2 h [14].

**Table 2:** Guidelines for diagnosis of GDM in China.

Guideline	Year	Screen Method	Diagnostic Criteria
Guideline for Clinical Diagnosis and Treatment of Gestational Diabetes Mellitus (draft) [32]	2007	Two Step: Step 1: 50 g GCT during 24–28 weeks of gestation; Step 2: 75 g or 100 g 3-h OGTT if the 1-h glucose value of 50 g GCT ≥ 7.8 mmol/L.	Two or more OGTT glucose values equal to or exceeds: Fasting: 5.3 mmol/L; 1 h: 10.0 mmol/L; 2 h: 8.6 mmol/L; 3 h 7.8 mmol/L.
-Diagnostic Criteria for Gestational Diabetes Mellitus (WS 331-2011) [29,30]	2011	One Step: 75 g OGTT	Any of the OGTT glucose values equal to or exceeds: Fasting: 5.1 mmol/L; 1 h: 10.0 mmol/L; 2 h: 8.5 mmol/L.
Guideline for Diagnosis and Treatment of Gestational Diabetes Mellitus (2014) [31]	2014	One Step: 75 g OGTT	Any of the OGTT glucose values equal to or exceeds: Fasting: 5.1 mmol/L; 1 h: 10.0 mmol/L; 2 h: 8.5 mmol/L.

**Table 3:** Risk factors for GDM.

	Risk Factors
Factors of mother	Advanced maternal age [12,13,22,33,34,35]
	Pre-pregnancy overweight or obesity [12,13,18,22,25,33,36]
	Excessive gestational weight gain [13,37,38]
	Dietary pattern [33,39,40,41]
	Cigarette smoking [13]
	Passive smoking [42]
	Parity [36]
History of disease	History of GDM [33,35,43,44]
	History of fetal macrosomia [36]
	Polycystic ovary syndrome [33,36,45,46,47]
Family history	Family history of diabetes [12,13,18,22,33,35,36,42,48]
Socioeconomic factor	Lower educational level [42]
	Lower understanding of health knowledge [42]
